# Sarcopenia and cognitive impairment after hip fracture in older adults: a narrative review and conceptual rehabilitation framework

**DOI:** 10.3389/fmed.2026.1955604

**Published:** 2026-09-28

**Authors:** Qi Han, Hong-zhao Bai

**Affiliations:** 1 Department of Rehabilitation Medicine, Panzhihua Central Hospital, Panzhihua, Sichuan, China; 2 Department of Rehabilitation Medicine, Zhejiang Dinghai Hospital (Zhoushan Branch of Ruijin Hospital, Shanghai Jiao Tong University School of Medicine), Zhoushan, Zhejiang Province, China; 3 Department of Rehabilitation Medicine, Hospital of Zhejiang People's Armed Police, Hangzhou, Zhejiang, China

**Keywords:** cognitive impairment, comprehensive geriatric assessment, delirium, hip fracture, rehabilitation, sarcopenia

## Abstract

Hip fracture in later life is followed by high mortality, incomplete recovery of mobility, and frequent loss of independence. Sarcopenia and cognitive impairment are common at presentation yet are often assessed and treated as separate determinants of recovery. This narrative review synthesizes evidence on their joint clinical impact, plausible links, and rehabilitation implications. In a 2025 rehabilitation cohort, independent ambulation was recovered by 60.8% of participants with both conditions versus 90.2% with neither; the combined phenotype was associated with a 57% lower likelihood of walking recovery. Recent studies also associate sarcopenia with postoperative delirium and early postoperative cognitive decline, whereas cognitive impairment is linked to more complications, less rehabilitation referral, and poorer function. Mechanistic translation remains incomplete: the FNDC5/irisin-BDNF axis has not been shown to mediate recovery in hip-fracture cohorts because target engagement, biomarker source, assay comparability, and perioperative confounding remain unresolved. We therefore propose an evidence-informed, phase-specific conceptual framework incorporating orthogeriatric assessment, delirium prevention, progressive resistance and task-specific exercise, individualized protein-energy support, cognitively adapted communication and motor learning, management of concerns about falling, and supported continuity after discharge. The framework integrates components supported by hip-fracture studies with recommendations extrapolated from broader geriatric guidance; it has not been validated as a complete care pathway for the combined phenotype. Future trials should include people with cognitive impairment, standardize sarcopenia definitions, measure rehabilitation exposure and adherence, and test whether targeting the combined phenotype improves patient-centered outcomes.

## 1 Introduction

Hip fracture is a sentinel event in later life. Many survivors do not regain prefracture mobility, and cognitive impairment is common and associated with complications, lower rehabilitation referral, and poorer outcomes (1–3). Sarcopenia and cognitive impairment are often treated as separate comorbidities, yet each may constrain effective rehabilitation: low muscle reserve limits safe intensity and repetition, whereas impaired attention, memory, or executive function can reduce learning, initiation, and generalization of movement strategies. Their coexistence therefore identifies a clinically vulnerable phenotype, although current observational evidence does not establish reciprocal causation.

This article is a focused narrative review that develops a conceptual, hypothesis-generating framework; it is not a systematic review. The proposed interaction model and phase-specific care framework should not be interpreted as a validated causal pathway or fixed multicomponent protocol. The objectives are to summarize the clinical burden of the combined muscle-cognition phenotype after hip fracture; distinguish established associations from candidate mechanisms; translate the evidence into a phase-specific rehabilitation framework; and define priorities for trials and implementation. The review treats sarcopenia as a co-primary phenotype, separates chronic cognitive impairment from delirium, integrates muscle quality and individualized nutritional support, and maps interventions across perioperative, acute inpatient, post-acute, and home phases. Figure 1 presents the proposed clinical model linking the combined phenotype to reduced effective rehabilitation dose and highlights modifiable rehabilitation leverage points.

**Figure 1:**
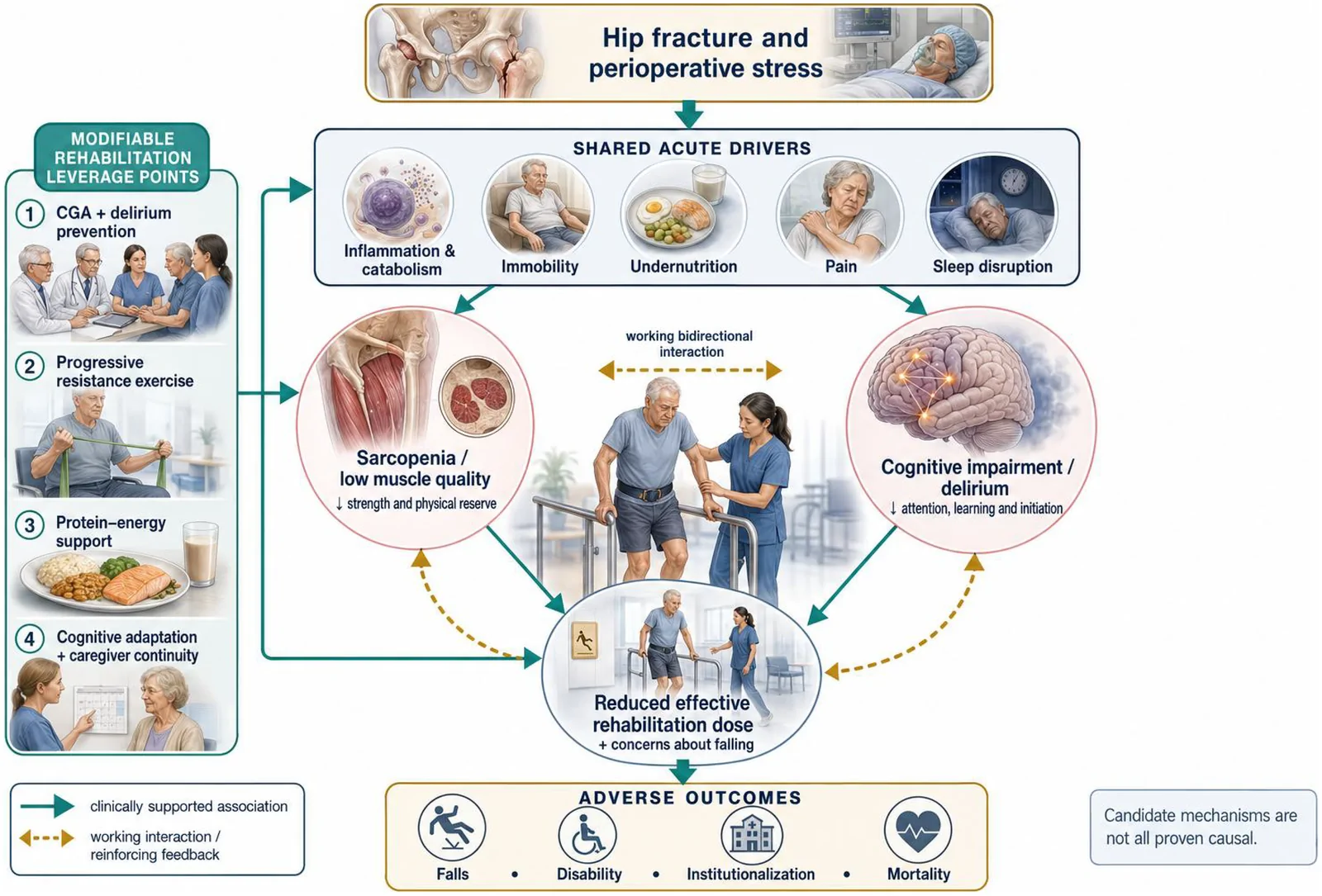
Original figure developed by the authors for this manuscript. Conceptual clinical model of coexisting sarcopenia and cognitive impairment after hip fracture and modifiable rehabilitation leverage points. The shared acute drivers are clinically observed, potentially modifiable exposures rather than a molecular causal chain. Solid arrows represent clinically supported associations; dashed arrows represent hypothesized interaction or reinforcing feedback. CGA, comprehensive geriatric assessment.

## 2 Review approach

This focused narrative review used a clinically oriented framework. PubMed and publisher websites were searched, and reference lists of eligible reviews and primary studies were checked. Searches were completed through 28 July 2026 and combined terms related to hip fracture with terms for sarcopenia, muscle strength, muscle quality, cognitive impairment, dementia, delirium, rehabilitation, resistance exercise, nutrition, fear of falling, kinesiophobia, and comprehensive geriatric assessment. Studies were eligible when they involved older adults with hip fracture or provided directly relevant geriatric evidence for one of the prespecified domains; reported data or guidance on muscle status, cognition or delirium, rehabilitation, nutrition, concerns about falling, or coordinated geriatric care; and were available as full-text English-language articles. Priority was given to consensus statements, systematic or scoping reviews, randomized trials, and prospective or retrospective hip-fracture cohorts published from 2019 onward. Earlier landmark guidance and trials were retained when they remained clinically relevant. Animal studies, case reports, conference abstracts, protocols without results, editorials without substantive evidence, studies unrelated to hip-fracture recovery, and duplicate reports were excluded. When reports described overlapping cohorts, the most complete or recent account was favored. Selection proceeded from title and abstract review to full-text assessment against the four review objectives. Candidate records were assessed for population relevance, study design, directness to hip fracture, and contribution beyond already included evidence. The reference list was then checked for coverage across the clinical phases and intervention domains. Each cited record was verified against a PubMed, publisher, or DOI record. Because this was a narrative rather than systematic review, screening was purposive, no protocol was registered, and no formal risk-of-bias or certainty-rating instrument was applied. Study estimates are therefore used to define the direction and limits of evidence, not to imply pooled causal effects or an exhaustive evidence set.

## 3 Clinical burden and evidence for interaction

### 3.1 The combined phenotype predicts impaired walking recovery

The most direct recent evidence comes from a 2025 rehabilitation cohort of 114 older adults after hip-fracture surgery. Independent ambulation was recovered by 90.2% of participants with neither sarcopenia nor cognitive impairment, compared with 60.8% of those with both. After adjustment, the combined group had a 57% lower likelihood of independent walking recovery (hazard ratio 0.431, 95% confidence interval 0.233–0.798) (4). This study is clinically compelling but observational and modest in size; residual confounding and setting-specific rehabilitation practices limit generalization. Additional studies support temporal links without establishing a complete reciprocal mechanism. Sarcopenia was associated with postoperative delirium in a geriatric hip-fracture cohort (5). A prospective study published in the 2026 volume of BMC Geriatrics associated preoperative sarcopenia with early postoperative cognitive decline (6). Cognitive impairment may also limit effective practice through difficulty understanding instructions, impaired error correction, low initiation, behavioral symptoms, or service exclusion. Direct evidence quantifying a reduction in the delivered or ‘usable’ rehabilitation dose is limited; this interpretation should therefore be treated as a clinical hypothesis. The 2025 cohort by Birkner and colleagues nevertheless showed that people with cognitive impairment were less likely to receive rehabilitation referral (2), supporting a care-delivery pathway that may amplify patient-level vulnerability.

### 3.2 Sarcopenia is common, but estimates depend on the definition

The Global Leadership Initiative in Sarcopenia (GLIS) defines sarcopenia conceptually as a disease of muscle characterized by compromised muscle health, while acknowledging that operational criteria remain heterogeneous (7). In clinical practice, regional consensus criteria remain necessary. The Asian Working Group for Sarcopenia (AWGS) 2019 uses low muscle strength, low muscle quantity or quality, and low physical performance, with handgrip-strength cutoffs of <28 kg for men and <18 kg for women (8). The revised European consensus (EWGSOP2) places low strength at the center of case finding and uses low muscle quantity or quality to confirm the diagnosis (9).Hip fracture complicates all three domains. Pain, traction, acute illness, weight-bearing restrictions, and fear can make gait speed or chair-stand testing invalid or unsafe. Edema and fluid shifts can distort bioelectrical impedance measurements. Computed tomography (CT) may provide opportunistic estimates of muscle attenuation or area, but acquisition level and segmentation methods vary. Consequently, a low grip-strength result during acute admission should be interpreted as clinically important low strength, not automatically as a fully confirmed chronic sarcopenia diagnosis. A defensible pathway is to screen early, document the constraints on testing, begin low-risk countermeasures, and confirm the phenotype once the patient is medically stable. A 2021 systematic review estimated that approximately 44% of older adults with hip fracture had sarcopenia, but diagnostic heterogeneity was substantial (10). A 2025 meta-analysis across fracture populations found that sarcopenia was associated with poorer overall prognosis; in the hip-fracture subgroup, it was associated with mortality, refracture, and especially poor functional recovery, although the magnitude and consistency of effects varied across outcomes (11). These data support routine case finding, while cautioning against presenting a single prevalence or prognostic estimate as universal.

### 3.3 Muscle strength and muscle quality contribute different information

Handgrip strength is inexpensive, repeatable, and feasible in many patients who cannot stand. It is therefore useful as a marker of global strength and recovery reserve. It should not, however, be treated as a stand-alone diagnostic or deterministic prognostic test. Grip strength can be lowered by pain, hand arthritis, neurologic disease, fatigue, and delirium; performance also depends on protocol and dynamometer position.

Longitudinal imaging studies show why serial assessment matters. Chen and colleagues reported ongoing loss of appendicular skeletal muscle after geriatric hip-fracture surgery, with greater loss and poorer functional recovery among patients with sarcopenia (12). Muscle quality may add information beyond size. In 679 older adults with hip fracture, Ge and colleagues found that gluteus medius/minimus muscle density, but not cross-sectional area, was independently associated with cognitive health (odds ratio 0.77, 95% confidence interval 0.62–0.96) (13). This association does not prove that myosteatosis causes cognitive impairment, but it supports the view that muscle composition and metabolic health may be more informative than mass alone.

### 3.4 Cognitive impairment, dementia, and delirium must be separated

“Cognitive impairment” in hip-fracture studies includes heterogeneous states: established dementia, previously unrecognized chronic impairment, transient effects of pain or medication, and delirium. These states have different time courses and treatment implications. Delirium is an acute disturbance of attention and awareness that fluctuates over hours or days. Dementia is a chronic decline that interferes with independence. They often coexist, and delirium can accelerate subsequent cognitive and functional decline (14).Screening should therefore answer two questions: “Is there an acute change?” and “What was the person's cognitive and functional baseline?” The 4 ‘A's Test (4AT) is a brief delirium screen validated in hospitalized older people (15). A positive screen requires clinical evaluation rather than being equated with dementia. Baseline cognition should be reconstructed from the patient, family or caregiver, primary-care records, medication history, and prefracture instrumental activities. Formal tools such as the Montreal Cognitive Assessment or Mini-Mental State Examination are more interpretable after pain, sleep, hearing, vision, acute illness, and delirium have been addressed.

## 4 Translational controversy: why the FNDC5/irisin-BDNF axis has not yet translated to hip-fracture rehabilitation

### 4.1 Biological promise exceeds population-specific evidence

The hypothesis is attractive: exercise-responsive PGC-1alpha/FNDC5 signaling may increase irisin, with downstream BDNF-related neuroplasticity providing a molecular bridge between contracting muscle and cognition. Experimental, translational, and general-aging studies support parts of this pathway, and recent molecular work confirmed that human FNDC5 can be translated from an upstream ATG codon and cleaved to produce an irisin precursor (16–21). However, no hip-fracture trial has shown that a change in irisin or BDNF mediates cognitive or mobility recovery. The current evidence chain is fragmented: animal target-engagement studies, biomarker associations in healthier populations, and clinical effects of exercise are often placed side by side as though they demonstrated one continuous causal sequence.

### 4.2 Source attribution and measurement remain unresolved

A peripheral concentration is not equivalent to central target engagement. Human skeletal muscle appears to express pro-BDNF rather than mature BDNF (22), while serum and plasma BDNF reflect different biological pools and are strongly influenced by clotting time, platelet content, and centrifugation (23). Commercial assays also differ in their ability to distinguish mature BDNF from pro-BDNF (24). Consequently, a perioperative rise or fall in circulating BDNF cannot be assumed to represent muscle secretion, blood-brain transport, or neuronal TrkB signaling. Irisin presents a parallel problem: molecular evidence supports human FNDC5 translation, but cleavage biology, reference ranges, and assay comparability remain insufficiently standardized for treatment selection (20, 21).

### 4.3 Hip fracture creates a particularly difficult translational test

The hip-fracture population differs from the animals and relatively healthy adults in whom the pathway is most persuasive. Tissue injury, anesthesia, blood loss or transfusion, acute inflammation, fasting, renal dysfunction, platelet changes, analgesic and sedative exposure, delirium, and abrupt immobilization occur simultaneously. These factors can alter both biomarker concentrations and the capacity to generate an exercise stimulus. Patients with the greatest cognitive and muscle deficits are also the least likely to complete demanding protocols and are frequently excluded from mechanistic studies. Sampling survivors who can exercise may therefore create an apparent biomarker-response relationship that does not generalize to the combined phenotype. Finally, short-term changes in a cognitive screening score during a delirium-prone admission cannot establish neuroplasticity and may reflect fluctuation, practice effects, pain control, or improved arousal.

### 4.4 What would constitute convincing translation?

A decisive study would embed mechanism within a hip-fracture rehabilitation trial rather than add a single biomarker sample to an observational cohort. It should prespecify serial sampling relative to surgery and exercise; use validated irisin assays and clearly defined serum or platelet-poor-plasma BDNF methods; record platelet count, transfusion, inflammation, renal function, nutrition, delirium, and medication exposure; quantify the rehabilitation dose actually completed; and test mediation of both mobility and cognitive outcomes. Inclusion of patients with cognitive impairment, supported consent, and longer follow-up are essential. Until such evidence exists, the FNDC5/irisin-BDNF axis should be treated as a hypothesis-generating pathway, not as a validated biomarker or therapeutic target for hip-fracture rehabilitation.

## 5 Translating assessment into treatment

Assessment should change care on the same day. Table 1 provides a minimal matrix. Table 2 summarizes the phase-specific sequencing, treatment emphasis, and progression or continuity criteria within the proposed integrated rehabilitation framework. A positive sarcopenia screen should trigger attention to progressive strengthening, protein–energy adequacy, and discharge continuity. Cognitive impairment should trigger adapted communication, repeated practice, environmental consistency, caregiver involvement, and delirium surveillance. A positive delirium screen should trigger evaluation of causes and a temporary adjustment—not abandonment—of rehabilitation goals.

**Table 1:** Decision-linked assessment of muscle, cognition, and modifiable rehabilitation barriers after hip fracture.

Domain	Initial trigger	Confirmation/monitoring	Decision linked to the finding
Muscle risk	Clinical case finding; grip strength when feasible; prefracture mobility and ADL	Repeat grip strength; calf circumference; DXA/BIA after fluid stabilization	Start individualized strengthening and nutrition review; confirm phenotype when stable
Chronic cognition	Patient/proxy history; prefracture IADL; brief screen when interpretable	Repeat after pain, sleep, sensory impairment, acute illness, and delirium improve	Adapt communication, repetition, environment, and caregiver-supported practice
Delirium	4AT plus assessment for acute change or fluctuation (15)	At least daily and after behavioral or functional change	Identify and treat causes; orient, mobilize, hydrate, protect sleep, and review medication
Nutrition	Recent intake, weight loss, swallowing, dentition, and feeding assistance	GLIM assessment if at risk (25); record actual intake and supplement consumption	Individualize energy/protein plan; coordinate feeding support and exercise
Function and safety	Bed mobility, transfers, sit-to-stand, walking, pain, and orthostatic symptoms	Repeat the same measure with assistance and cueing documented	Increase successful task practice or investigate barriers before progression
Concerns about falling	Identify avoided activity and reason; FES-I for fall concern; TSK for pain-related movement fear	Repeat against actual exposure to the avoided task	Use graded exposure, analgesia, feedback, self-efficacy goals, and proportionate assistance
Discharge context	Home/care setting, caregiver capacity, food access, technology, transport, and equipment	Reassess before every transition	Assign follow-up ownership and provide a progression and contingency plan

**Table 2:** Phase-specific sequencing and progression within the proposed integrated rehabilitation framework.

Phase	Primary aim	Phase-specific treatment emphasis	Progression or continuity criterion
Perioperative/0–72 h	Prevent avoidable decline and detect delirium	Orthogeriatric/CGA review; pain and delirium prevention; hydration and nutrition; short bouts of bed mobility, transfer, sit-to-stand, and walking	Progress when medically stable and the task is reproducibly safe with documented assistance
Acute inpatient	Build effective practice and protect intake	Daily task-specific mobility; individualized progressive strengthening; balance and gait practice; nutrition support when indicated	Increase load or task complexity one variable at a time; track completed repetitions, cueing, and symptoms
Post-acute rehabilitation	Restore independence and confidence in real environments	Higher-volume gait and ADL practice; progressive resistance; graded exposure for avoided tasks; caregiver practice	Add dual-task or environmental challenge only after the single motor task is stable and safe
Home/long-term care	Sustain progression and reduce falls, readmission, and nutritional decline	Supervised home exercise, walking and environment-specific practice; adherence support; bone-health and fall-prevention plan	Use a simple written/visual plan, defined follow-up owner, and telehealth only when access and assistance are adequate

Individual components are supported to differing degrees by hip-fracture studies and broader geriatric guidance. The combined package and its sequence have not been validated specifically in patients with coexisting sarcopenia and cognitive impairment.

Phenotyping is most useful when it avoids false precision. In the first 24–48 h, classification into low strength or muscle risk, cognitive vulnerability, active delirium, nutrition risk, and concerns about falling is usually more actionable than a definitive subtype. Measures can be refined after stabilization, and repeated measures are preferable because pain, fatigue, fluid status, and attention change rapidly. The framework has three evidence layers: direct hip-fracture evidence, quantitative targets extrapolated from broader geriatric guidance, and a conceptual integration of sequence and coordination. The relevant component evidence is appraised in Sections 6 and 7; the combined package has not been validated as a protocol.6. A phase-specific integrated rehabilitation framework.

### 5.1 Perioperative and immediate postoperative phase

The first objectives are to prevent avoidable decline and create conditions for participation. Orthogeriatric co-management or early comprehensive geriatric assessment should address pain, hydration, oxygenation, anemia where clinically relevant, bowel and bladder problems, medication burden, sleep, sensory aids, nutrition, pressure injury, thrombosis prevention, bone health, and discharge planning. Multicomponent delirium prevention includes orientation, family contact, hearing and visual aids, sleep protection, hydration, early mobilization, and avoidance of unnecessary deliriogenic medication.

Mobilization should begin as soon as medically and surgically feasible, with weight-bearing status confirmed. The early exercise “dose” may consist of bed mobility, transfers, sit-to-stand practice, short bouts of walking, and low-load activation of quadriceps and gluteal muscles. A systematic review found that exercise initiated within the first three months improves physical function, but heterogeneity prevented identification of a superior protocol (26). The practical priority is frequent, task-specific practice without allowing fatigue, orthostatic symptoms, uncontrolled pain, or delirium to turn a single unsuccessful session into prolonged inactivity.

Cognitively adapted delivery begins immediately. Use one-step commands, demonstration before verbal explanation, consistent terminology, short sessions, repetition, and visible goals. Check hearing aids and glasses. For a person with delirium, schedule activity during lucid periods and use familiar caregivers when possible. Safety can be increased by close supervision and environmental simplification rather than by defaulting to bed rest.

### 5.2 Inpatient rehabilitation: progressive strength, task practice, and nutrition

Progressive resistance training is the principal muscle-directed intervention, but the dose should match healing, surgical precautions, cardiovascular tolerance, cognition, and baseline reserve. Hip-fracture studies support progressive strengthening across several functional outcomes, yet small samples and protocol heterogeneity do not establish one optimal frequency, load, or repetition range (27, 28). Early sessions can use low-load knee extension, hip abductor activation, repeated sit-to-stand, supported heel raises, and step practice. As tolerance improves, a target such as two or three sessions per week, one to three sets, and approximately 8–12 repetitions at moderate effort may be considered. These numerical targets are pragmatic extrapolations from broader older-adult resistance-training principles rather than a validated prescription for patients with both hip fracture, sarcopenia, and cognitive impairment. They are progression goals, not mandatory starting doses.

Strength training should be embedded in meaningful tasks: rising from the toilet, transferring to a chair, turning, reaching, and walking to a personally relevant destination. Balance and gait practice should include the assistive device and environment expected at discharge. Dual-task training can be useful later, but only after the primary motor task is safe and reasonably stable. Introducing a cognitive task too early in a patient with delirium, severe attentional impairment, or unstable gait can reduce movement quality and increase risk. Nutrition is not an optional add-on. Screening should occur on admission, followed by etiologic and phenotypic assessment when malnutrition is suspected. The Global Leadership Initiative on Malnutrition framework requires at least one phenotypic and one etiologic criterion for diagnosis (25). The ESPEN geriatric guideline suggests approximately 30 kcal/kg/day as a general energy guide and 1.2–1.5 g protein/kg/day during acute or chronic illness (29). These values come from broader geriatric guidance, not trials establishing an optimal intake for the combined sarcopenia–cognitive impairment phenotype after hip fracture. They must be individualized for actual intake, body size, renal function, disease severity, swallowing safety, tolerance, and goals of care. Hip-fracture-specific evidence more directly supports targeted oral nutritional supplementation for patients with inadequate intake or malnutrition risk, although the certainty and outcome profile remain variable (30, 31).Evidence for oral nutritional supplements (ONS) is supportive but should not be overstated. A Cochrane review found low-quality evidence that multinutrient oral supplementation started before or soon after surgery may reduce complications, without a clear mortality effect (30). A 2023 meta-analysis of randomized trials found reductions in infective complications, pressure ulcers, total complications, and length of stay, while also identifying adherence as an important limitation (31). ONS should therefore be targeted to inadequate intake or malnutrition risk, monitored for actual consumption, and integrated with exercise rather than prescribed as a substitute for rehabilitation.

### 5.3 Cognitive adaptation and motor learning

People with cognitive impairment can benefit from rehabilitation, but the method of delivery matters. A Cochrane review found uncertainty about the best enhanced rehabilitation model for dementia after hip-fracture surgery, largely because trials were small and heterogeneous (32). A scoping review nevertheless identified modifiable features across settings: interdisciplinary care, early mobilization, structured exercise, staff training, environmental support, and caregiver involvement (33).

Practical adaptations include:

- one instruction at a time, using the same cue words across disciplines;
- demonstration, guided movement, errorless learning, and high repetition;
- spaced retrieval of safety steps and assistive-device use;
- visible orientation cues and a consistent therapy location when possible;
- goals tied to familiar roles and activities;
- brief sessions distributed through the day when sustained attention is limited;
- caregiver education focused on cueing and safe practice, not only supervision.

These adaptations change how motor training is delivered; they do not replace strength, balance, or gait training. A 2025 narrative review of hip-fracture rehabilitation in cognitive impairment similarly emphasized integrated rather than exclusionary care (34). Treatment decisions should be based on observed capacity, goals, and response to supported practice—not a cognitive-screen cutoff alone.

### 5.4 Concerns about falling and pain-related kinesiophobia

Fear of falling and kinesiophobia are related but not interchangeable. Fear of falling concerns anticipated falls and their consequences. Kinesiophobia is excessive fear of movement or reinjury, usually conceptualized in relation to pain. The Tampa Scale for Kinesiophobia, Falls Efficacy Scale–International, and fear-of-falling measures should therefore not be treated as if they assess the same construct. A 2023 systematic review found that concerns about falling after hip fracture were common and consistently associated with poorer physical function; among reviewed measures, the FES-I and revised Fear of Falling Questionnaire had hip-fracture validation (35). Tool selection should therefore follow the clinical question. FES-I is appropriate for concern about falling during activities; TSK is more defensible when pain-related movement fear is the target. Scores should be paired with a short interview to identify the feared activity, expected harm, pain behavior, prior falls, and confidence. Intervention evidence is mixed. The FIT-HIP cluster randomized trial combined cognitive-behavioral techniques and graded exposure but did not show superiority for its primary fear-of-falling outcomes (36). The “Step by Step” intervention improved fall-related self-efficacy and physical performance but did not increase daily walking duration (37). These results argue for targeted, behaviorally informed treatment rather than assuming that education alone or a generic protocol will change activity. Clinically, graded practice should be anchored to a specific avoided task, adequate analgesia, achievable goals, feedback on success, and progressive reduction of unnecessary assistance.

### 5.5 Discharge and post-acute continuity

Losses accumulate when supervised rehabilitation stops before strength, confidence, and daily routines are restored. Discharge planning should specify the exercise progression, nutritional plan, cognitive cueing strategy, assistive device, caregiver role, red flags, and follow-up responsibility. Home hazards, transportation, medication management, food access, and caregiver capacity are part of the rehabilitation prescription. A multicomponent home program improved selected functional outcomes after hip-fracture surgery in a randomized trial (38). More specifically, a 2026 randomized trial in 60 older adults with mild cognitive impairment after hip fracture found that a six-month home-based Otago exercise program reduced fall risk and improved selected cognitive and frailty outcomes (39). The sample was small, so replication is needed, but the trial demonstrates that cognitive impairment does not preclude structured home exercise. Telerehabilitation may support monitoring and continuity when used selectively. A 2025 systematic review and meta-analysis found uncertain effects, with small improvements in fall efficacy but no clear clinically important improvement in activities of daily living (40). Remote delivery should therefore supplement, not automatically replace, hands-on assessment for patients with sensory impairment, severe cognitive impairment, low digital access, or high physical-assistance needs.

## 6 Comprehensive geriatric assessment as the coordinating platform

Comprehensive geriatric assessment (CGA) is a multidimensional, interdisciplinary process that links assessment to a coordinated treatment and follow-up plan. Its value in hip fracture is organizational as much as diagnostic: it creates a shared account of medical stability, function, cognition, mood, nutrition, medication, continence, falls, social support, and discharge barriers. The Trondheim Hip Fracture Trial showed that comprehensive geriatric care delivered in a geriatric ward improved mobility and reduced costs compared with usual orthopedic care (41). A Cochrane review of older people admitted to surgical services found that CGA probably increased the chance of being alive and living at home, although evidence across specific outcomes varied (42). A 2025 hip-fracture systematic review found predominantly favorable associations between early CGA and patient-reported and clinical outcomes, but heterogeneity precluded a single pooled effect and limited causal interpretation (43).CGA should not become a long checklist that delays action. It should produce a same-day problem list and ownership map. For the combined muscle–cognition phenotype, five decisions are especially important:

1. Is acute delirium present, and what reversible contributors require treatment?
2. What is the patient's current strength and muscle-risk profile, and which measures can be repeated?
3. Is intake adequate for the planned rehabilitation dose?
4. Which communication and learning adaptations will make practice effective?
5. What support is required to continue the program after discharge?

An integrated rehabilitation trial provides proof of concept for coordinated delivery. In the FIRM randomized trial, a multidisciplinary pathway including rehabilitation, geriatric management, and discharge continuity improved mobility-related outcomes over one year compared with conventional rehabilitation (44). A 2026 prospective cohort further suggested that nutritional status modifies the association between rehabilitation strategy and functional recovery, with greater benefit of integrated rehabilitation among malnourished participants (45). Because this latter study was observational, it should be interpreted as hypothesis-generating rather than evidence for a nutrition-by-treatment causal interaction.

## 7 Implementation principles

### 7.1 Treat rehabilitation exposure as a measurable intervention

‘Received rehabilitation’ is too imprecise. Studies and services should record minutes, repetitions, intensity, task type, missed sessions, reasons for nonparticipation, cueing required, and practice outside formal therapy. Here, a successful, task-relevant practice repetition is defined operationally as one completed or purposefully assisted attempt at a predefined functional task—such as sit-to-stand, transfer, turning, or walking—performed with acceptable safety and movement quality under the least cueing and physical assistance appropriate to the patient's current level. Reporting should include the number of attempts, proportion completed, cueing and assistance level, limiting symptoms, and adverse events. This is a proposed measurement construct rather than a validated metric. Cognitive impairment may reduce the effective practice achieved within a scheduled session, but direct evidence quantifying this effect is limited; the concept should be tested prospectively rather than assumed.

### 7.2 Use repeated, decision-linked measures

Minimal repeated measures could include grip strength when feasible, a transfer or sit-to-stand measure, walking level, a delirium screen, an intake record, and a patient-reported measure of concerns about falling. The purpose is not surveillance for its own sake. Failure to improve should trigger a search for untreated pain, delirium, infection, anemia, orthostatic intolerance, low intake, depression, fear, inappropriate assistance, or insufficient progression.

### 7.3 Include, rather than systematically exclude, cognitive impairment

Trials often exclude dementia, severe cognitive impairment, or inability to provide conventional consent, producing evidence least applicable to those at highest risk. Services can reproduce the same inequity when cognitive impairment is used to deny intensive rehabilitation. Supported consent, proxy involvement, accessible communication, caregiver participation, and outcomes that can be observed reliably are necessary for both inclusive research and equitable practice.

### 7.4 Minimum viable intervention when multidisciplinary resources are limited

Where geriatricians, dietitians, psychologists, or daily physiotherapy are unavailable, the essential functions can be organized around one accountable coordinator rather than an entire co-located team. A nurse, rehabilitation assistant, or other trained staff member can use a brief daily bundle: check for acute cognitive change, pain or orthostatic symptoms, intake, and mobility; deliver several short, supervised bouts of transfer, sit-to-stand, and walking practice within surgical precautions; use consistent one-step cues, demonstration, orientation, and sensory aids; and prompt protein-energy intake or feeding assistance according to the prescribed nutrition plan. Family caregivers and nursing assistants can reinforce this bundle after competency-based teaching and teach-back, using a simple pictorial checklist that records completed repetitions, cueing or assistance, intake, and warning signs (15, 31–33).

Delegation requires explicit boundaries. The initial mobility and weight-bearing plan and the stop criteria should be set by an available clinician; new delirium, chest pain, syncope, a new neurologic deficit, wound concern, uncontrolled pain, or sudden functional loss should trigger escalation. Specialist input can be referral-based or remote rather than continuous. This minimum viable intervention is not equivalent to comprehensive multidisciplinary care; it is a pragmatic floor intended to prevent default bed rest, missed feeding, inconsistent cueing, and exclusion from rehabilitation when staffing is constrained.

### 7.5 Avoid universal prescriptions

Protein targets, exercise intensity, number of sessions, and technology are not one-size-fits-all. Renal disease, dysphagia, heart failure, severe behavioral symptoms, fracture stability, pain, prefracture function, and goals of care modify the plan. The integrated framework is an evidence-informed coordination model assembled from components with differing levels of directness; it is not a rigid or validated multicomponent protocol.

## 8 Research priorities

The evidence base has four major gaps. First, only a small number of studies directly test the combined sarcopenia–cognitive impairment phenotype. Large, multicenter cohorts should use harmonized sarcopenia definitions, distinguish chronic impairment from delirium, and model interaction on an additive as well as multiplicative scale.

Second, intervention trials should test components and sequence. Pragmatic factorial or adaptive designs could compare standard rehabilitation with cognitively adapted delivery, progressive resistance, and individualized nutrition, while measuring whether effects differ by baseline muscle and cognitive status. Trials should report treatment fidelity, actual ONS consumption, and the amount of successful practice.

Third, mechanistic trials should apply the design requirements outlined in Section 4.4 and determine whether standardized biomarker changes mediate patient-centered mobility and cognitive outcomes.

Fourth, post-discharge research should address caregiver burden, adherence, access, and sustainability. The key question is not whether a supervised program works under ideal conditions, but how older adults with cognitive impairment can continue safe, progressive activity and adequate nutrition in real homes and care facilities.

## 9 Strengths and limitations of this review

This review extends prior cognitive-impairment-focused syntheses by treating sarcopenia as a co-primary phenotype; incorporating direct 2024–2026 evidence on walking recovery, delirium, cognition, and muscle quality; separating delirium from chronic cognitive impairment; distinguishing fear of falling from pain-related kinesiophobia; and linking assessment to phase-specific treatment decisions. It also makes the directness of evidence explicit by distinguishing hip-fracture data, broader geriatric guidance, and conceptual integration. The review is narrative and used purposive rather than exhaustive study selection; it did not register a protocol, use duplicate systematic screening, apply a formal risk-of-bias instrument, or grade certainty. The literature is heterogeneous in sarcopenia definitions, cognitive measures, rehabilitation settings, and outcomes. Many mechanistic claims derive from experimental or general aging research rather than hip-fracture cohorts. Several recent studies are single-center or modest in size, and people with severe cognitive impairment remain underrepresented. Accordingly, the proposed framework is evidence-informed and clinically testable, but neither the combined interaction model nor the full care pathway has been prospectively validated.

## 10 Conclusion

Sarcopenia and cognitive impairment frequently coexist after hip fracture and jointly identify patients at high risk of incomplete recovery. Treating them as a combined clinical phenotype is justified for risk stratification and care planning, not yet as a proven biological syndrome. The most defensible evidence concerns prognosis and modifiable barriers to treatment; the proposed reciprocal biology remains hypothesis-generating.
